# *Lactobacillus acidophilus *ameliorates *H. pylori*-induced gastric inflammation by inactivating the Smad7 and NFκB pathways

**DOI:** 10.1186/1471-2180-12-38

**Published:** 2012-03-19

**Authors:** Yao-Jong Yang, Ching-Chun Chuang, Hsiao-Bai Yang, Cheng-Chan Lu, Bor-Shyang Sheu

**Affiliations:** 1 Department of Pediatrics, National Cheng Kung University Hospital, College of Medicine, National Cheng Kung University, Tainan, Taiwan; 2 Department of Pathology, National Cheng Kung University Hospital, College of Medicine, National Cheng Kung University, Tainan, Taiwan; 3 Department of Internal Medicine, National Cheng Kung University Hospital, College of Medicine, National Cheng Kung University, Tainan, Taiwan; 4 Institute of Clinical Medicine, National Cheng Kung University Hospital, College of Medicine, National Cheng Kung University, Tainan, Taiwan; 5 Department of Pathology, Ton-Yen General Hospital, Hsin-Chu County, Taiwan

## Abstract

**Background:**

*H. pylori *infection may trigger Smad7 and NFκB expression in the stomach, whereas probiotics promote gastrointestinal health and improve intestinal inflammation caused by pathogens. This study examines if probiotics can improve *H. pylori*-induced gastric inflammation by inactivating the Smad7 and NFκB pathways.

**Results:**

Challenge with *H. pylori *increased IL-8 and TNF-α expressions but not TGF-β1 in MKN45 cells. The RNA levels of Smad7 in AGS cells increased after *H. pylori *infection in a dose-dependent manner. A higher dose (MOI 100) of *L. acidophilus *pre-treatment attenuated the *H. pylori*-induced IL-8 expressions, but not TGF-β1. Such anti-inflammatory effect was mediated via increased cytoplasmic IκBα and depletion of nuclear NFκB. *L. acidophilus *also inhibited *H. pylori*-induced Smad7 transcription by inactivating the Jak1 and Stat1 pathways, which might activate the TGF-β1/Smad pathway. *L. acidophilus *pre-treatment ameliorated IFN-γ-induced Smad7 translation level and subsequently reduced nuclear NF-κB production, as detected by western blotting.

**Conclusions:**

*H. pylori *infection induces Smad7, NFκB, IL-8, and TNF-α production *in vitro*. Higher doses of *L. acidophilus *pre-treatment reduce *H. pylori*-induced inflammation through the inactivation of the Smad7 and NFκB pathways.

## Background

*Helicobacter pylori *infection is considered a major factor inducing chronic gastritis, peptic ulcer, and even gastric cancer in humans [1-3]. In mice and human studies, the gastric mucosa of *H. pylori*-infected subjects show up-regulated NF-κB pathway and Th1 type cytokine responses [4-9], which may disturb the integrity of the gut epithelial barrier [10]. Accordingly, the inactivation of the NF-κB pathway and its downstream immune cascades may be helpful in preventing serious *H. pylori*-induced complications.

Probiotics are known to inhibit enteric pathogens likes *Salmonella, Shigella*, and *Citrobacter rodentium *in both *in vitro *and animal models [11-13]. Their potential clinical benefits in preventing or resolving gastrointestinal diseases have been emphasized [14,15]. There are several mechanisms through which they provide gut protection, including decreasing the luminal pH value by producing lactic acid [16,17] or by competing with gut pathogens for host surface receptors [18].

Nonetheless, Coconnier et al. have shown that probiotics may inhibit *H. pylori *growth independent of pH and lactic acid levels [19] while Tien et al. report that *Lactobacillus casei *may down-regulate *Shigella flexneri*-induced pro-inflammatory cytokines, chemokines, and adherence molecules by inhibiting the NF-κB pathway [12]. Another critical mechanism involving probiotics relates to changes in host immune responses to infection via reduced TNF-α and IL-8 but increased IL-10 [20,21]. Regarding the brief contact between the flora of probiotics and the gastric epithelium, an intake of probiotics by *H. pylori*-infected patients has anti-inflammation benefits resulting from a change in host immune responses.

Transforming growth factor (TGF)-β1 negatively regulates Th1 cell such that TGF-β1/deficient mice spontaneously develop gastritis [22,23]. It is well accepted that the TGF-β1 signaling pathway is positively regulated by receptor-associated Smad 2/3, but negatively by Smad7 [24,25]. *H. pylori *infection is reportedly associated with increased expression of gastric Smad7, but controversial results in TGF-β1 levels [26,27]. These suggest that the TGF-β1/Smad signaling pathway plays an important role in gut inflammation. However, the exact mechanism of probiotics reducing *H. pylori*-induced gastric inflammation remains unclear. Thus, this study aimed to examine whether probiotics could regulate the Smad- and NFκB-mediated signaling pathways to reduce the down-stream inflammatory cytokine production after *H. pylori *infection.

## Methods

### Cell lines and culture condition

This study was approved by the Ethical Committee of National Cheng Kung University Hospital (ER-98-208). Two human gastric epithelial cancer cell lines (MKN45 and AGS) were obtained from the Health Science Research Resources Bank in Japan and maintained in RPMI 1,640 medium (GIBCO BRL, Grand Island, NY) and F-12 medium (GIBCO BRL, Grand Island, NY) containing 10% FBS at 37°C in a humidified atmosphere (95%) with 5% CO_2_. The cells were sub-cultured every second day. Prior to the bacterial infection study, the cells were incubated in antibiotic-free RPMI 1,640 medium containing 10% FBS overnight at 37°C in 5% CO_2_.

### Bacteria and culture condition

Bacterial strain (HP238) isolated from a clinical patient was used. The HP238 expressed CagA, VacA, and BabA proteins in previous studies [28,29]. The bacteria were maintained on a Brucella agar plate containing 10% horse serum and incubated under micro-aerophilic conditions (10% CO_2_, 5% O_2 _and 85% N_2_) for 24-48 hours. The bacteria were then transferred to PBS before infecting the cells. Growth density was measured spectrophotometrically at 600 nm. The infectious dose of bacteria was 1 × 10^8 ^bacteria/ml at an OD of 1.

The MKN45 cells were infected with a multiplicity of infection (MOI) 1-100 for various time periods. A probiotic strain, one contained in AB-yogurt, *Lactobacillus acidophilus *(LA5^®^, originated from the Chr. Hansen, Denmark, provided by the President Corp., Tainan, Taiwan) was used. The bacteria were maintained on a Brucella agar, incubated in anaerobic conditions, and then harvested and suspended in phosphate-buffered saline (PBS) before infection. The viable density of *L. acidophilus *was 1 × 10^8 ^bacteria/ml at an OD of 1.

### MKN45 cells viability after exposure to *H. pylori *and *L. acidophilus*

The cytotoxicity of MKN45 cell exposure to *H. pylori *and *L. acidophilus *was determined by percentage of lactate dehydrogenase (LDH) leakage (Cytotoxicity Assay, Promega Co., Madison, WI, USA) and by assessing viable cell counts using non-stained trypan blue. The culture supernatant and remaining MKN45 cells were collected after incubation with variable doses (MOI 1-1000) of *L. acidophilus *and *H. pylori *(MOI 100) for 8 and 4 hours, respectively. Cell cultures without bacterial infection served as controls. The procedures were performed according to the instruction manuals and post-infection cells with non-stained trypan blue staining were directly counted.

### Enzyme-linked immuno-sorbent assay (ELISA) for cytokines

To determine the optimal dose and incubation time of various bacteria, bacteria (*H. pylori *and *L. acidophilus*) were cultured with MKN45 cells (MOI 1-100) in an antibiotic-free RPMI 1,640 medium (5 ml) containing 10% FBS at 35°C in micro-aerophilic conditions for up to 8 hours. In the experimental study, *L. acidophilus *were added to MKN45 cells and incubated for 8 hours under the same conditions. After PBS washing and removal of the bacilli, an equal volume of *H. pylori *was added and the cells were incubated for another 4 hours. The final culture supernatant was centrifuged at 12,000 rpm for 5 min to remove bacteria and cell debris. Concentrations of TNF-α, IL-8 (R & D System, Minneapolis, MN), and TGF-β1 (eBioscience, San Diego, CA) were measured by ELISA according to the manufacturer's instructions. The absorbance of each micro-plate was read on a spectro-photometer using 450 nm as the primary wave length and 570 nm as the reference wave length. All tests were done in triplicate.

### Preparation of cytoplasmic and nuclear extracts

The MKN45 and AGS cells were pre-treated with *L. acidophilus *for 8 hours followed by various doses of *H. pylori *for 1 hour; then cytoplasmic and nuclear extracts were isolated by a Nuclear Extract Kit (Active Motif, Japan). Briefly, cells were washed with ice-cold saline containing phosphatase inhibitors and pelleted. The cell pellets were then re-suspended in a hypotonic buffer and incubated for 15 min on ice. They were lysed by the addition of detergent and vortexed vigorously for 10 s. After the nuclei were pelleted and re-suspended in complete lysis buffer, the tube was vigorously shaken at 4°C for 30 min on a shaking platform. The nuclear extracts were then centrifuged and the supernatants were aliquoted and stored at -80°C.

### RT-PCR for cytoplasmic Smad7

Total RNA was isolated from MKN45 cells using a commercial kit (ImProm-ll™ Reverse Transcription System, Promega, USA) after *H. pylori *and *L. acidophilus *incubation. The RNA was quantified by determining absorbance at 260 nm. One *μ*g RNA was converted to cDNA, which was stored at -72°C until use. The human Smad7 primer sequences were forward 5'-CATCACCTTAGCCGACTCTG-3' and reverse 5'GTCTTCTCCTCCCAGTATGC-3', generating a 224 bp fragment [30]. For Jak1 and Stat1, the primer sequences were forward 5'-GCAGCCAGCATGATGAGA-3' and 5'-GTGGACGAGGTTTTGTAAGGA-3' and reverse 5'-CTCGGAAGAAAGGCCTCTG-3' and 5'-CAGACACAGAAATCAACTC-3', generating fragments of 607 bp and 518 bp, respectively [31,32]. The PCR condition was as follows; 95°C for 5 min, followed by 25 cycle of 95°C for 1 min, 56°C for 1 min, and 72°C for 1 min, and finally 72°C for 7 min. The primer sequences for human β-actin was forward 5'-GTCTTCCCCTCCATCGTG-3' and reverse 5'-GTCATCTTCTCGCGGTTG-3', generating a 272 bp fragment. The amplification conditions were as follows: 95°C for 5 min, then a 20 cycle of 95°C for 1 min, 50°C for 1 min, 72°C for 1 min, and 72°C for 7 min.

### Western blotting for NF-κB, IκB-α and Smad7

Interferon gamma (IFN-γ) (PeproTech Inc., NJ, USA) 50 μl (100 ng/ml) was added to each dish in the experimental studies. The cytoplasmic and nuclear extracts were washed with ice-cold PBS and lysed in a 0.5 ml/well lysis buffer (150 mmol/l NaCl, 20 mmol/l Tris, pH 7.5, 0.1% Triton X-100, 1 mmol/l phenylmethylsulfonyl fluoride [PMSF] and 10 μg/ml aprotonin) as modified from the reports of Kim et al. and Moon et al. [33,34]. Protein concentrations in the lysates were determined using the Pierce BCA Protein Assay Kit (Thermo scientific, USA). Protein/lane 10 μg was then size-fractionated into a denaturing, non-reducing 10% polyacrylamide minigel and electrophoretically transferred to polyvinylidene fluoride (PVDF) (0.45-μm pore size) (Millpore Corparation, USA).

Specific proteins were detected using rabbit antihuman NF-κB p65, rabbit anti-human IκB-α (1:1000, Cell Signaling, Boston, MA, USA), and mouse anti-human Smad7 (1:500, R&D System, USA, MN) as primary antibodies, and peroxidase-conjugated anti-rabbit IgG, anti-mouse IgG (1:10000) as a secondary antibody. Specifically bound peroxidase was detected by Chemiluminescent HRP Substrate (ECL system, Millpore Corparation, USA) and then exposed to x-ray (GE Healthcare, UK) for 10-30 s.

### Statistical analysis

The Student's *t *test and paired *t *test were used, as appropriate, for parametric differences. One-way analysis of variance (ANOVA) with Bonferroni's correction was applied for the multiple testing of data. The Mann-Whitney *U *test was used for the difference between non-parametric data while Pearson's *χ*2 test was used for non-parametric proportion difference. All tests were two-tailed and a *P *< 0.05 was considered statistically significant.

## Results

### Cell viability after incubation with *H. pylori *and *L. acidophilus*

The cytotoxicity and viability of MKN45 cells incubated with *H. pylori *(MOI 100) and *L. acidophilus *(MOI 1-1000) were determined by assessing the percentage leakage of LDH and non-stained trypan blue at the 4^th ^and 8^th ^hours, respectively (Table 1). Plasma membrane damage assessed by the percentage of LDH leakage from MKN45 after *H. pylori *incubation (18.1%) was not different to those of control cells (18.0%). Moreover, the viable cell count calculated by non-stained trypan blue did not markedly decrease. When *L. acidophilus *was incubated with MKN45 cells for 8 hours, the cytotoxicity and viable cell count at MOI 1-100 were not significantly affected. However, LDH leakage and cell death slightly increased as incubation with MOI 1,000 for 8 hours. Therefore, the optimal dose of bacteria used for the experimental study was limited to MOI 100.

**Table 1:** Cell cytotoxicity and viable cell counts of MKN45 after co-incubation with *H. pylori *and *L. acidophilus *determined by the percentage of LDH leakage (in triplicate) and non-stained trypan blue (single)

Bacteria and MOI	Cytotoxicity^a ^(% LDH)	Viable cell count (× 10^6^)
Cell only for 4 and 8 hours	18.0, 18.0	1.36
*H. pylori *for 4 hours		
MOI 100	18.1	1.00
*Lactobacillus *for 8 hours		
MOI 1	18.4	1.00
MOI 10	18.0	1.11
MOI 100	18.7	1.24
MOI 1000	24.2	0.77

^a^All cytotoxicity data were presented with mean value of three tests

### *H. pylori *stimulated IL-8 and TNF-α but not TGF-β1 production *in vitro*

**Figure 1:**
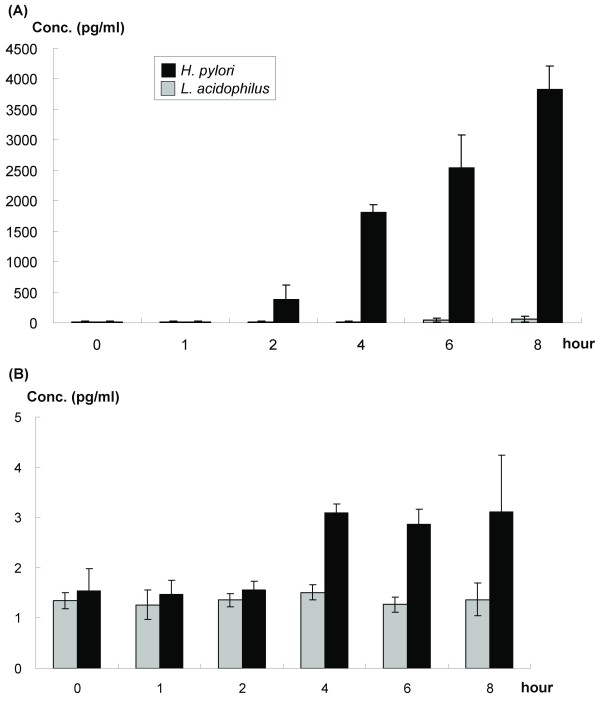
**(A) IL-8 and (B) TNF-α concentrations in the supernatant of MKN45 cells culture after variable duration of *H. pylori *and *L. acidophilus *infection (MOI = 100)**. Data were expressed as means ± standard deviation (SD) (in triplicate).

In MKN45 cells incubated with *H. pylori *(MOI 100) at various time periods, the IL-8 level increased from the 4^th ^to the 8^th ^hour after co-incubation, as determined by ELISA (Figure 1A). For TNF-α, the post-incubation level rose after the 4^th ^hour and maintained a plateau until the 8^th ^hour (Figure 1B). However, the TGF-β1 level did not increase after *H. pylori *incubation for 4 hours (data not shown).

In contrast, *L. acidophilus *did not induce IL-8, TNF-α, and TGF-β1 expressions of MKN45 at least within the 8-hour co-incubation period.

### Pre-treatment of *L. acidophilus *attenuated *H. pylori-induced *IL-8

**Figure 2:**
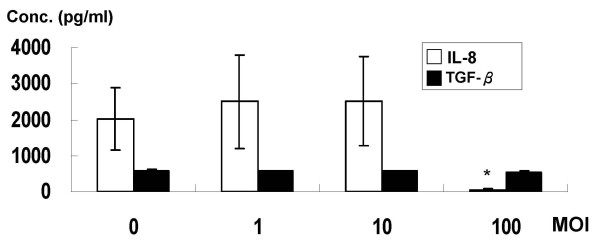
**The concentrations of IL-8 (blank column) and TGF-β1 (black column) in the supernatant of MKN45 cells pre-treated with different MOI (0: control; 1: 1 × 10^6 ^c.f.u.; 10: 1 × 10^7 ^c.f.u.; 100: 1 × 10^8 ^c.f.u.) of *L. acidophilus***. The cells were washed thrice with PBS to remove the *L. acidophilus *and then infected with *H. pylori *(MOI = 100) for 4 hours. Data are expressed as means ± SD (in triplicate). Statistical analysis was performed in each measurement with comparisons to the controls (cells treated *H. pylori *only; IL-8 2034 ± 865 pg/ml and TGF-β1 587.2 ± 39.8 pg/ml) (*P < 0.05).

Because the IL-8 level of MKN45 cells could be induced by *H. pylori *challenge for 4 hours, the time- and dose-dependent effects of probiotics in reducing pro-inflammatory cytokines and TGF-β1 on the 4^th ^hour were studied. The IL-8 and TGF-β1 concentrations were shown for MKN cells challenged by *H. pylori *and with variable doses of *L. acidophilus *pretreatment for 8 hours (Figure 2). Compared to the control group, *L. acidophilus *pre-treatment with higher bacterial colony count (MOI 100) reduced *H. pylori*-induced IL-8 expressions in MKN45 cells (*P *< 0.05). The TGF-β1 level did not change (*P *> 0.05).

### *L. acidophilus *reduced *H. pylori-induced *NF-κB by increasing IκBα

**Figure 3:**
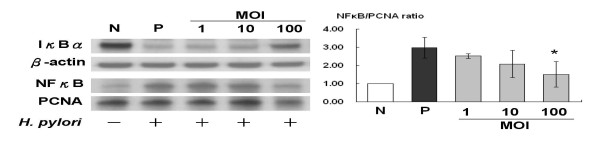
**The IκBα and NFκB expressions after various doses of *L. acidophilus *pretreatment for 8 hours followed by *H. pylori *co-incubation for 1 hour**. N, MKN45 cell only; P, *H. pylori*, 1 × 10^8 ^c.f.u. treatment for 1 hour; MOI 1, pre-treatment with *L. acidophilus *1 × 10^6 ^c.f.u. for 8 hours followed by *H. pylori *treatment for 1 hour; MOI 10, *L. acidophilus *1 × 10^7 ^c.f.u. followed by *H. pylori *treatment for 1 hour; MOI 100, *L. acidophilus *1 × 10^8 ^c.f.u. followed by *H. pylori *treatment for 1 hour (*P < 0.05).

The study determined that MKN45 cells (MOI 100) incubated with *H. pylori *led to a peak increase of nuclear NF-κB production within one hour. Thus, nuclear NF-κB levels of MKN45 cells co-incubated with *H. pylori*, after prior pre-treatments by various MOIs (1-100) of *L. acidophilus *were tested for anti-inflammatory effects of probiotics. Pre-treatment of *L. acidophilus *increased cytoplasmic IκBα but decreased the nuclear NF-κB levels induced by *H. pylori *in a dose-dependent manner (Figure 3). Because IκBα level could be mediated by activating the TGF-β1/Smad signaling pathway, the role Smad7 played in *L. acidophilus *restoring TGF-β1/Smad activity after *H. pylori *challenge was tested.

### *L. acidophilus *inhibited *H. pylori-*and IFN-γ-induced Smad7 expression

**Figure 4:**
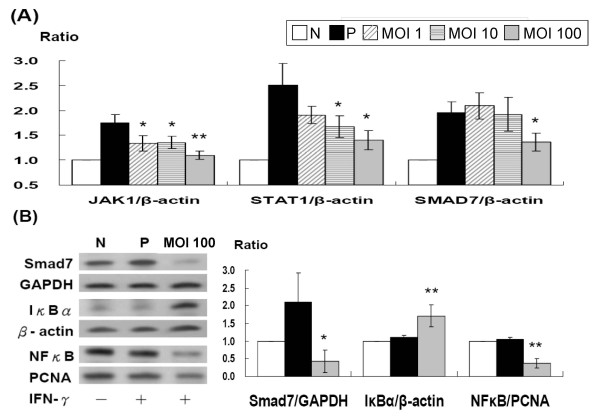
**Pre-treatment of *L. acidophilus *significantly reduced JAK1 (MOI 1-100), STAT1 (MOI 10-100), and SMAD7, and subsequent NFκB production after (A) *H. pylori *and (B) IFN-γ treatment**. N, AGS cell only; P, *H. pylori*, MOI = 100 (A, black column) and 100 ng/ml IFN-γ (B, black column) treatment for 0.5 hour; MOI 1, 10, and 100 meant pre-treatment with *L. acidophilus *1 × 10^6^, 1 × 10^7^, 1 × 10^8 ^c.f.u. for 8 hours, respectively, followed by *H. pylori *treatment for 0.5 hour (* *P *< 0.05; ** *P *< 0.01).

The Figure 4A shows that pre-treatment with high-dose *L. acidophilus *(MOI 100) for 8 h prevented *H. pylori*-induced Smad7 production by semi-quantitative RT-PCT. Compared to positive controls (AGS cells co-incubated with *H. pylori *at MOI 100), *L. acidophilus *pretreatment as high as MOI 100 significantly reduced the *H. pylori*-induced Smad7 production at the RNA level (*P *< 0.05) via inactivation of Jak1 and Stat1 transcriptions. *L. acidophilus *pre-treatment also inhibited the expression of IFN-γ-induced Smad7 protein (*P *< 0.05) *in vitro*, with a subsequent increase in cytoplasmic IκBα (*P *< 0.01) and a decrease in nuclear NF-κB (*P *< 0.01) (Figure 4B).

## Discussion

Human immunity plays an important role in the development of more serious clinical diseases after *H. pylori *infection because of increased pro-inflammatory cytokine expressions on the patients' gastric mucosa [6,8]. *H. pylori *infection can activate NF-κB in gastric epithelium cells and subsequently up-regulate IL-8 gene transcription [4]. Consistent with previous human studies [6-9], the present study reveals that *H. pylori *infection can induce TNF-α and IL-8 pro-inflammatory cytokine expressions *in vitro*. In agreement with the animal study reported by McCarthy et al. [35], the present study illustrates that yogurt-containing probiotics, *L. acidophilus *does not stimulate pro-inflammatory cytokines after an 8-hour incubation with MKN45 cells. This suggests that probiotics can exert anti-inflammatory effects *in vitro*. Accordingly, it will be interesting to test how the inflammatory cascades can be counteracted by probiotics.

The antimicrobial activity of *Lactobacillus *against enteric pathogens is, in part, due to the accumulation of lactic acid [17,21]. The ability of lactic acid production varies in the *Lactobacillus spp*. and *L. acidophilus *is a low lactic acid-production strain [34]. Experimentally, *L. acidophilus *decreases the viability of *H. pylori in vitro *independent of pH and lactic acid levels [19]. The pH value of each suspension in this study is around 6.8-7.0 (data not shown). Other mechanisms like immuno-modulation should therefore contribute largely to the anti-inflammatory effects of *L. acidophilus*.

The current study demonstrates that *L. acidophilus *pre-treatment can decrease the *H. pylori*induced nuclear NF-κB expression in the 1^st ^hour and IL-8 in the 4^th ^hour, after co-culture with *H. pylori *and MKN45 cells. Furthermore, the TNF-α level is also decreased although its value is quite low (data not shown). This study further confirms that such suppression occurs in a dose-dependent manner and is mediated through the stabilization of IκBα. The finding is compatible with the results of Tien et al. showing that anti-inflammatory effects can only be achieved at an adequate bacteria count in probiotics [12]. Data from the present study indicate that *L. acidophilus *can counteract *H. pylori*-induced gastric inflammation specifically by mediation through the IκBα/NF-κB pathway in a dose-dependent manner.

**Figure 5:**
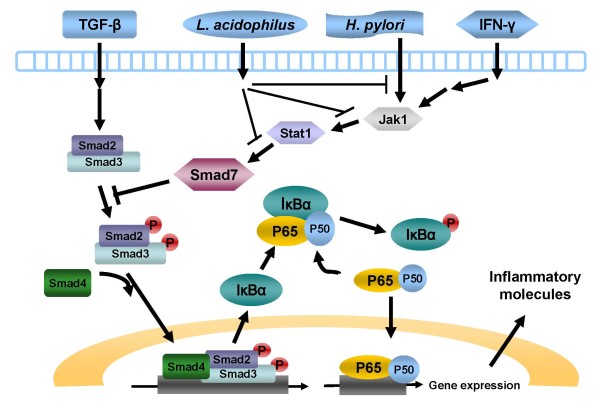
**Schematic diagram to illustrate possible pathways of *L. acidophilus *inhibition of *H. pylori*-induced inflammation on gastric epithelium through TGF-β/Smad3, IFN-γ/Smad7, and NFκB signals**.

In normal intestinal mucosal cells, the TGF-β1 signal may negatively regulate NF-κB activation by stimulating the negative regulator, IκBα [36]. *H. pylori *infection reportedly may inhibit the TGF-β1 signal pathway via activation of the gastric Smad7 expression [26]. This study also declares that both *H. pylori *and *L. acidophilus *do not affect the TGF-β1 production of gastric epithelial cells, which again confirm that *L. acidophilus *regulates TGFβ1/Smad3 downstream activity by restoring Smad7. The present study is the first to demonstrate that *L. acidophilus *can down-regulate Smad7 production to restore the TGFβ1/Smad activity and to ameliorate the *H. pylori*-induced gastric inflammation *in vitro *(Figure 5).

Smad7 can also be induced in normal gastric specimens by IFN-γ through a STAT1 dependent pathway [26]. In fact, the gastric epithelium does not secret IFN-γ. Therefore, *H. pylori *(up-regulation) and *L. acidophilus *(down-regulation) both significantly regulates Smad7 in epithelium cells through the mediation of the STAT1-dependent Smad7 pathway. Inhibiting Smad7 can restore the TGF-β1/Smad3 signaling and result in the suppression of inflammatory cytokine production in patients with inflammatory bowel diseases [37,38]. The data here reveals that probiotics contained in yogurt can inhibit Smad7 to diminish *H. pylori*related gastric inflammation. Such probiotics can be quite promising for the improvement of *H. pylori *infection control.

## Conclusions

Yogurt-containing *L. acidophilus *can improve *H. pylori*-induced gastric inflammation through the inactivation of the Smad7 and NF-κB mediated pathways. Intake of *L. acidophilus-*containing yogurt may improve gastric inflammation in *H. pylori*-infected patients.

## Authors' contributions

YYJ conducted this study and wrote the first manuscript. CCC correlated the sera of subjects and performed the tests. YHB and LCC gave suggestions for the interpretation of results, while SBS provided the critical revision of the manuscript and reviewed the statistical analysis. All authors read and approved the final manuscript.
